# Supplementary material for: Benchmarking Deep Learning Architectures for Predicting Readmission to the ICU and Describing Patients-at-Risk
Source: Sci Rep. 2020 Jan 24;10:1111. doi: 10.1038/s41598-020-58053-z (PMC6981230; doi:10.1038/s41598-020-58053-z)
Supplement: Supplementary file 1 — Supplementary Information. [file 41598_2020_58053_MOESM1_ESM.pdf]

Supplementary Information

Supplementary Table S1. Baseline characteristics of the analysed intensive care unit (ICU) stays.

	**Readmission Within 30 Days**	**No Readmission Within 30 Days**
	N=5,495	N=39,803
**Num. Recent Admissions** (Mean [Range])	0.7 [0-23]	0.3 [0-12]
**ICU Length of Stay** (Days) (Mean [Range])	5 [0-117]	4 [0-173]
**Pre-ICU Length of Stay** (Days) (Mean [Range])	3 [0-141]	2 [0-155]
**Age** (Years) (Mean [Range])	65 [18-100]	63 [18-103]
**Gender** Male	3136 (57.1 %)	22536 (56.6 %)
**Admission Location**		
Emergency Room Admit	2537 (46.2 %)	17251 (43.3 %)
Clinic Referral / Premature	1086 (19.8 %)	7950 (20.0 %)
Transfer from Hospital / Extramural	1011 (18.4 %)	6546 (16.4 %)
Phys Referral / Normal Delivery	797 (14.5 %)	7843 (19.7 %)
Transfer from Skilled Nursing Facility	44 (0.8 %)	168 (0.4 %)
Other / Unknown	20 (0.4 %)	45 (0.1 %)
**Ethnicity**		
White	3974 (72.3 %)	28508 (71.6 %)
Black / African American	679 (12.4 %)	3806 (9.6 %)
Other / Unknown	489 (8.9 %)	4523 (11.4 %)
Hispanic / Latino	174 (3.2 %)	1462 (3.7 %)
Asian	116 (2.1 %)	920 (2.3 %)
Unable to Obtain	63 (1.1 %)	584 (1.5 %)
**Insurance**		
Medicare	3328 (60.6 %)	20850 (52.4 %)
Private	1487 (27.1 %)	13596 (34.2 %)
Medicaid	550 (10.0 %)	3676 (9.2 %)
Government	109 (2.0 %)	1222 (3.1 %)
Self-Pay	21 (0.4 %)	459 (1.2 %)
**Marital Status**		
Married / Life Partner	2604 (47.4 %)	19215 (48.3 %)
Single	1511 (27.5 %)	10471 (26.3 %)
Widowed / Divorced / Separated	1182 (21.5 %)	8248 (20.7 %)
Other / Unknown	198 (3.6 %)	1869 (4.7 %)
**Elective Surgery Admission** Yes	513 (9.3 %)	5729 (14.4 %)
